# Variant of Hoffa disease: a case report

**DOI:** 10.51866/cr.819

**Published:** 2025-06-24

**Authors:** Sharifah Nor Amirah Syed Abdul Latiff Alsagoff, Mohd Fairudz Mohd Miswan, Mohd Yusoff Yahaya, Mohamed Faizal Sikkandar, Anis Safura Ramli

**Affiliations:** 1 MD, MS (Orth), Department of Orthopaedic Surgery and Traumatology, Faculty of Medicine, Universiti Teknologi MARA (UiTM), Sungai Buloh, Selangor, Malaysia. E-mail: drfairudz@yahoo.com, fairudz@uitm.edu.my, drfairudz@gmail.com.my; 2 MBBS, MS (Orth), Department of Orthopaedic Surgery and Traumatology, Faculty of Medicine, Universiti Teknologi MARA (UiTM), Sungai Buloh, Selangor, Malaysia.; 3 MBBS, MS (Orth), Department of Orthopaedic Surgery and Traumatology, Faculty of Medicine, Universiti Teknologi MARA (UiTM), Sungai Buloh, Selangor, Malaysia.; 4 MBBS, MS (Orth), Department of Orthopaedic Surgery and Traumatology, Faculty of Medicine, Universiti Teknologi MARA (UiTM), Sungai Buloh, Selangor, Malaysia.; 5 MBBS, MRCGP, FRCGP, CPE JCPTGP, DFFP, DRCOG, Department of Primary Care, Faculty of Medicine, Universiti Teknologi MARA (UiTM), Sungai Buloh, Selangor, Malaysia.

**Keywords:** Anterior knee pain, Chronic Knee pain, Hoffa fat pad

## Abstract

This report presents the case of an 18-year-old female student with a low BMI, who was otherwise healthy. She presented with chronic anterior right knee pain persisting for 1 year. The pain began spontaneously and gradually increased in severity, especially during long distance walking and standing for more than 15 minutes. The symptoms did not subside with medication and physiotherapy. All biochemical and radiological investigations to rule out other related possible aetiologies were unremarkable. The patient eventually underwent an arthroscopic knee surgery, during which the intraoperative findings revealed the Hoffa fat pad over the anteromedial knee joint. This was debrided and after surgery, she was symptom free and had resumed her normal activities.

## Introduction

Knee pain affects approximately 25% of the adult population and its prevalence has increased by almost 65% over the past 20 years.^1,2^ This results in nearly 4 million primary care visits annually.^3^ Primary care physicians should conduct an initial evaluation that includes comprehensive history taking and a systematic physical examination to exclude urgent causes and assess the need for referral.^3^

Regarding investigations, radiographic imaging should be reserved for patients with chronic knee pain lasting more than 6 weeks or those with acute traumatic pain. Other imaging modalities such as musculoskeletal ultrasonography and magnetic resonance imaging (MRI) can be considered for detailed evaluation. Laboratory tests can be used when a particular cause such as infection is suspected.^3^

For patients with chronic knee pain, analgesia and physical therapy are the mainstay treatments, particularly when all investigation findings are normal, excluding specific knee pathologies. The goal of physiotherapy is to restore the biomechanics via muscle strengthening,^4^ to achieve a supple knee range of motion and maintain proprioception. However, referral to orthopaedic surgeons is indicated if patients remain symptomatic despite conservative management.

One of the rare differential diagnoses in patients with chronic anterior knee pain is Hoffa pad impingement syndrome, also known as Hoffa disease. This condition involves the infrapatellar fat pad, an extra synovial but intracapsular structure that occupies most of the anterior knee compartment.^5^ Impingement of the infrapatellar fat pad between the patella and distal femoral condyle may result in pain. In this report, we present the case of a young female student with chronic right knee pain, who was diagnosed with a variant of Hoffa disease.

## Case presentation

An 18-year-old female university student presented to a primary care clinic a year ago with chronic right knee pain, associated with reduced range of movement. She described the initial pain as gradual in onset involving an unaccustomed long-distance walk, which started following a festival celebration. A few days later, she developed moderate pain on standing and walking, which worsened during flexion and stairs activity. However, she remained asymptomatic when her knee was in an extended position while lying supine. In addition, the patient denied any relevant family history or symptoms suggestive of systemic arthritic-related diseases such as systemic lupus erythematosus.

Since then, the patient had multiple visits to both public and private primary care clinics as well as a hospital emergency department. She was prescribed several types of oral and topical analgesics for pain control and had multiple sessions of physiotherapy. However, to no avail, she remained symptomatic.

The patient was subsequently referred to the orthopaedic outpatient clinic by her primary care physicians for further assessment and management.

On physical examination, she was thin built with a low BMI. She walked with a normal gait and had normal body posture. Right knee examination revealed normal skin appearance with minimal swelling over the medial joint compartment, which was not warm to touch. There was mild tenderness at the inferomedial aspect of the patella and medial joint line. Special assessments for ligament laxity and meniscus injury revealed normal findings. The patient demonstrated normal knee motion although mild anterior knee pain was elicited on terminal extension. Therefore, clinically, she was suspected to have synovial impingement syndrome and plica syndrome.

Her radiographic findings were normal. MRI was conducted, which revealed a grade 1 posterior horn medial meniscus injury. This finding did not tally with the physical findings, nor did it support the provisional diagnosis. All blood investigations to rule out other causes of arthralgia or arthritis were conducted yielding normal results. These included the full blood count with differentials, erythrocyte sedimentation rate, C-reactive protein level, rheumatoid factor level, antinuclear antibody level and serum uric acid level.

**Figure 1:**
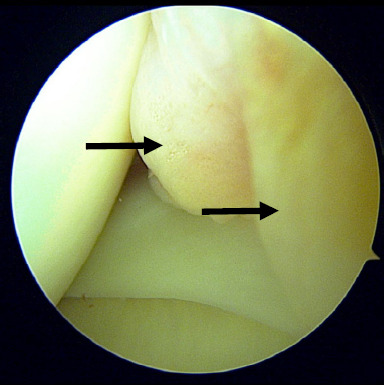
Thickened and inflamed synovium at the anteromedial joint.

**Figure 2:**
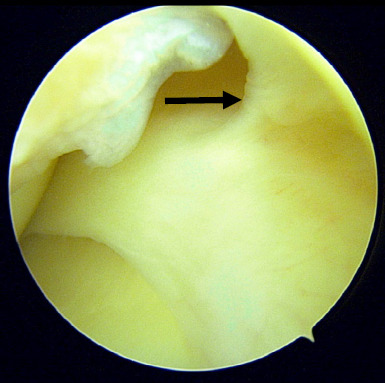
Thickened capsule at the meniscal body extending superiorly.

**Figure 3:**
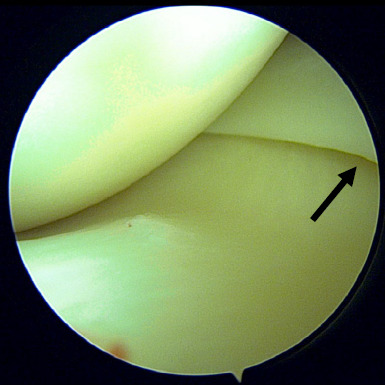
Meniscal body slightly elevated due to a retraction effect.

Due to persistent symptoms and failure of conservative treatment modalities, the patient was counselled for surgical intervention particularly diagnostic knee arthroscopic surgery. Intra-operative findings showed thickened and inflamed synovium tissue in the anteromedial compartment (Figure 1) and suprapatellar area. However, this tissue was not impinged during knee extension and flexion. After removal of the synovium, a thick band on the capsule of the body of the medial meniscus was identified (Figure 2). This band was persistent in its position and caused elevation of the body of the meniscus during knee flexion and extension (Figure 3). We strongly believe that this was the primary cause of her persistent pain as all other structures including the meniscus, appeared normal. After debridement and release of the abnormal tissue, the position of the meniscus returned to normal. Additionally, five tissue samples were taken from all compartments, and histopathology examination revealed benign synovial tissue with fibrosis.

A few months after surgery, the patient remained asymptomatic, gained full knee function and resumed her normal daily activities.

## Discussion

Several differential diagnoses should be considered in young adults presenting with chronic anterior knee pain. Isolated anterior knee pain suggests involvement of either the patella, quadriceps or patellar tendon and its attachment.

A dull type of anterior knee pain, exacerbated by engaging in prolonged activity or climbing stairs, is commonly associated with patellofemoral syndrome.^6^ Adults engaged in sports with repetitive overuse can develop patellar tendinopathy or quadriceps tendinopathy known as jumper’s knee.

Another differential diagnosis for anterior knee pain in young adults is Hoffa pad impingement syndrome, also referred to as Hoffa disease, fat pad impingement or infrapatellar fat pad syndrome. This condition is often successfully treated with conservative modalities such as physiotherapy. In some cases, arthroscopic debridement of the fat pad has been shown to provide a favourable outcome.^7^

The Hoffa fat pad is generally known to occupy the infra-retro aspect of the patellar tendon.^8^ There are four plicae named according to their relation to the patella: infrapatellar, suprapatellar, medial and lateral patellar.^9^ This fat pad is well-vascularised and innervated.^10^ It is one of the many causes of anterior knee pain. This normal tissue becomes symptomatic when it becomes inflamed and results in an impingement, known as Hoffa fat pad syndrome. This inflammatory process can cause thickening and fibrosis leading to the loss of the elastic properties of the normal infrapatellar fat pad.^9^ The scar tissue eventually accumulates and impinges at the intercondylar notch, causing painful extension.^11^ The development of inflammation, fibrosis and pain within the fat pad is largely due to the interactions between fibroblast, vascular endothelial growth factors, tumour necrosis factor and interleukin-6.^12^

The Hoffa test should be performed to reproduce the symptom when the abovementioned diagnosis is suspected. This is conducted by applying firm pressure with the thumb just inferior to the patella along either side of the patella tendon in 30-degree knee flexion. Pain is elicited as soon as the knee is brought to full extension.^13^ A positive test result and a loss of extension and flexion are the usual symptoms of anterior interval scarring. Other symptoms include discomfort during extension and anterior knee pain.

The epidemiology of Hoffa fat pad impingement is unknown, which could be due to the difficulty in diagnosing the condition and the need for treatment with surgery. On MRI, the Hoffa fat pad appears similar to subcutaneous adipose tissue.^14^ Sagittal MRI is the gold standard imaging modality for assessing Hoffa fat pad pathology. The foci of lower-signal intensity would represent the interposed fibrous septae.^15^

Regarding management, physical therapy including muscle strengthening may improve patellar tracking. This focuses on closed-chain exercises targeting the quadriceps and the vastus medialis oblique muscle. Surgical intervention is warranted when patients have exhausted conservative treatment. Arthroscopic debridement is the primary surgical option. A symptomatic infrapatellar fat pad has been demonstrated to respond well to arthroscopic excision. Approximately 86%-91% of patients experience few to no post operative complaints.^16^ Evidence has shown that knee arthroscopic surgery has a low complication rate of 1.1%, although it can increase up to 5% in high-volume surgical centres.^17^

In this case, the patient did not present with the classical symptoms of Hoffa pad syndrome. She was diagnosed to have a variant of Hoffa fat impingement which resulted in thickening of the capsule wall in the form of a band extending from the anterior horn of the medial meniscus to the inferior pole of the patella. She was successfully treated with arthroscopic debridement of the thick band on the medial meniscus.

## Conclusion

Hoffa pad syndrome is a diagnosis of exclusion as multiple conditions can cause anterior knee pain and extension block or pseudo locking. A positive Hoffa test result is the most reliable finding that suggests this diagnosis. Other intra-articular pathologies such as plica syndrome, meniscus tears and osteochondral injuries must also be ruled out. Primary care physicians play a pivotal role in monitoring patients presenting with knee pain and ensuring early referral to orthopaedic clinics when patients remain symptomatic. Arthroscopic debridement in patients diagnosed with Hoffa fat impingement can greatly alleviate symptoms and enhance patient satisfaction.
